# Supplementary material for: Effects of Age, Diet CP, NDF, EE, and Starch on the Rumen Bacteria Community and Function in Dairy Cattle
Source: Microorganisms. 2021 Aug 23;9(8):1788. doi: 10.3390/microorganisms9081788 (PMC8400643; doi:10.3390/microorganisms9081788)
Supplement: Supplementary file 1 [file microorganisms-09-01788-s001.zip › microorganisms-1338572-suppl-conversion.pdf]

Supplementary Material

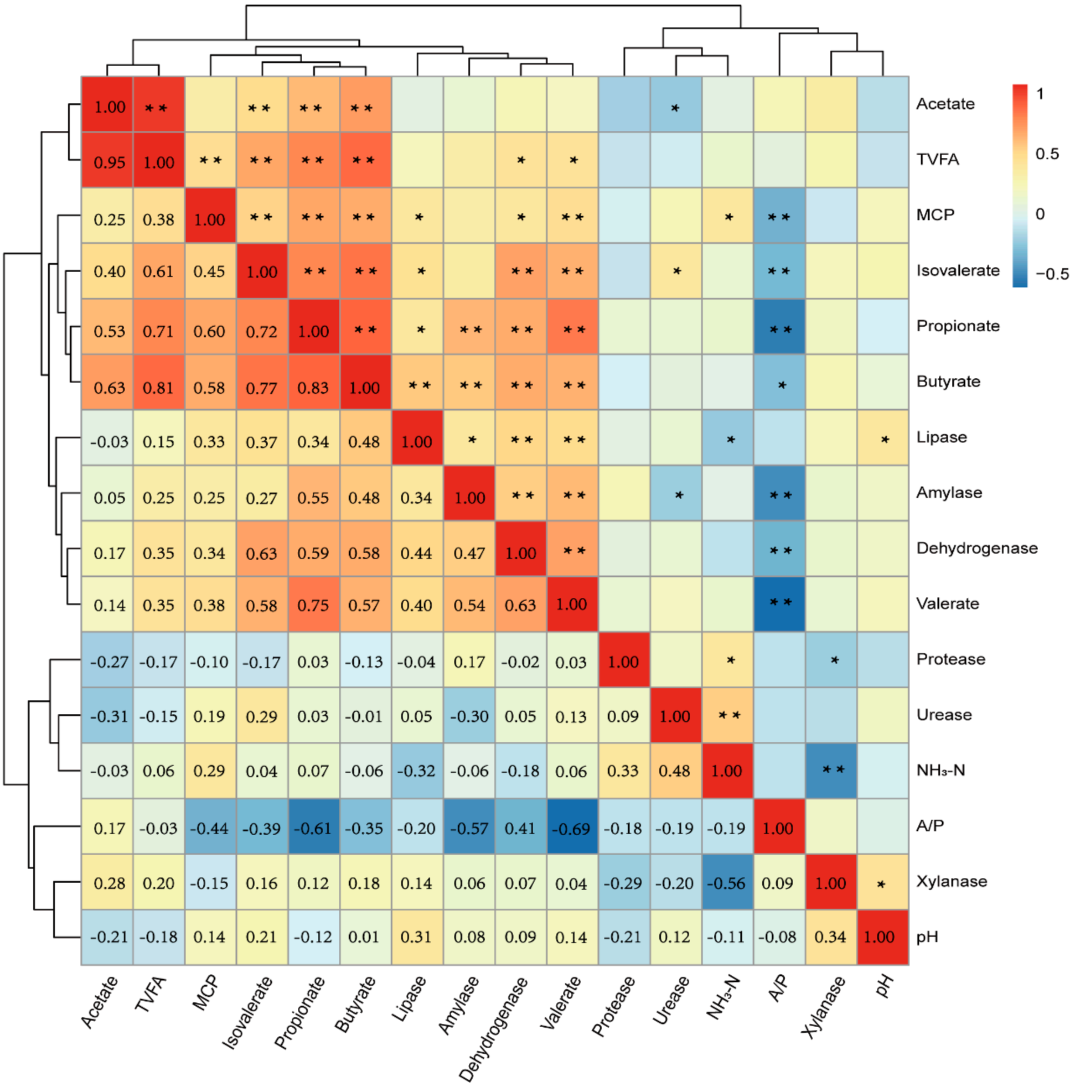

Figure S1. The correlation between enzyme activity and rumen fermentation profile. Cells are colored based on Spearman's correlation coefficient: red represents a positive correlation, and blue represents a negative correlation. "*," "**," indicate FDR adjusted *P*-values <0.05, and <0.01, respectively. NH_3-N: ammonium nitrogen; MCP: microbial crude protein; TVFA: total volatile fatty acid; A/P: the ratio of acetate to propionate.

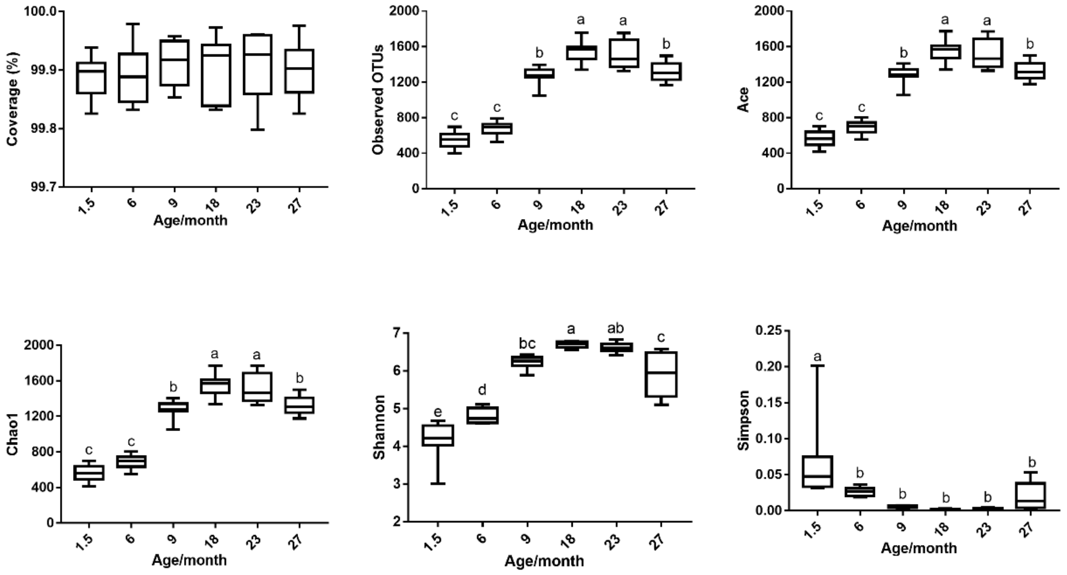

Figure S2. Comparison of the rumen bacteria α-diversity of different ages of dairy cattle. The different letters mean that the difference is significant ($P < 0.05$).

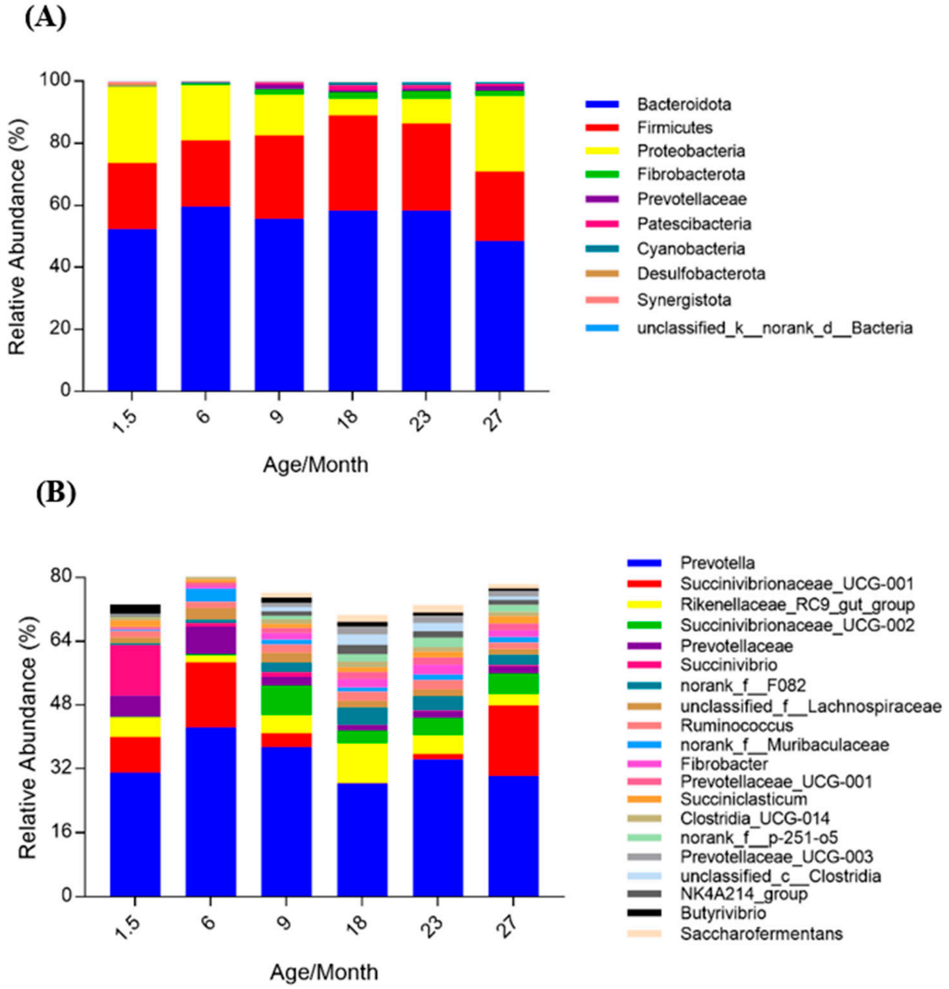

Figure S3. Histograms of the relative abundances of rumen microbial taxa at different ages. (**A**) At the phylum level (top ten). (**B**) At the genus level (top twenty). The X-axis shows the ages of the experimental dairy cattle, and the Y-axis represents the relative abundances of bacteria.

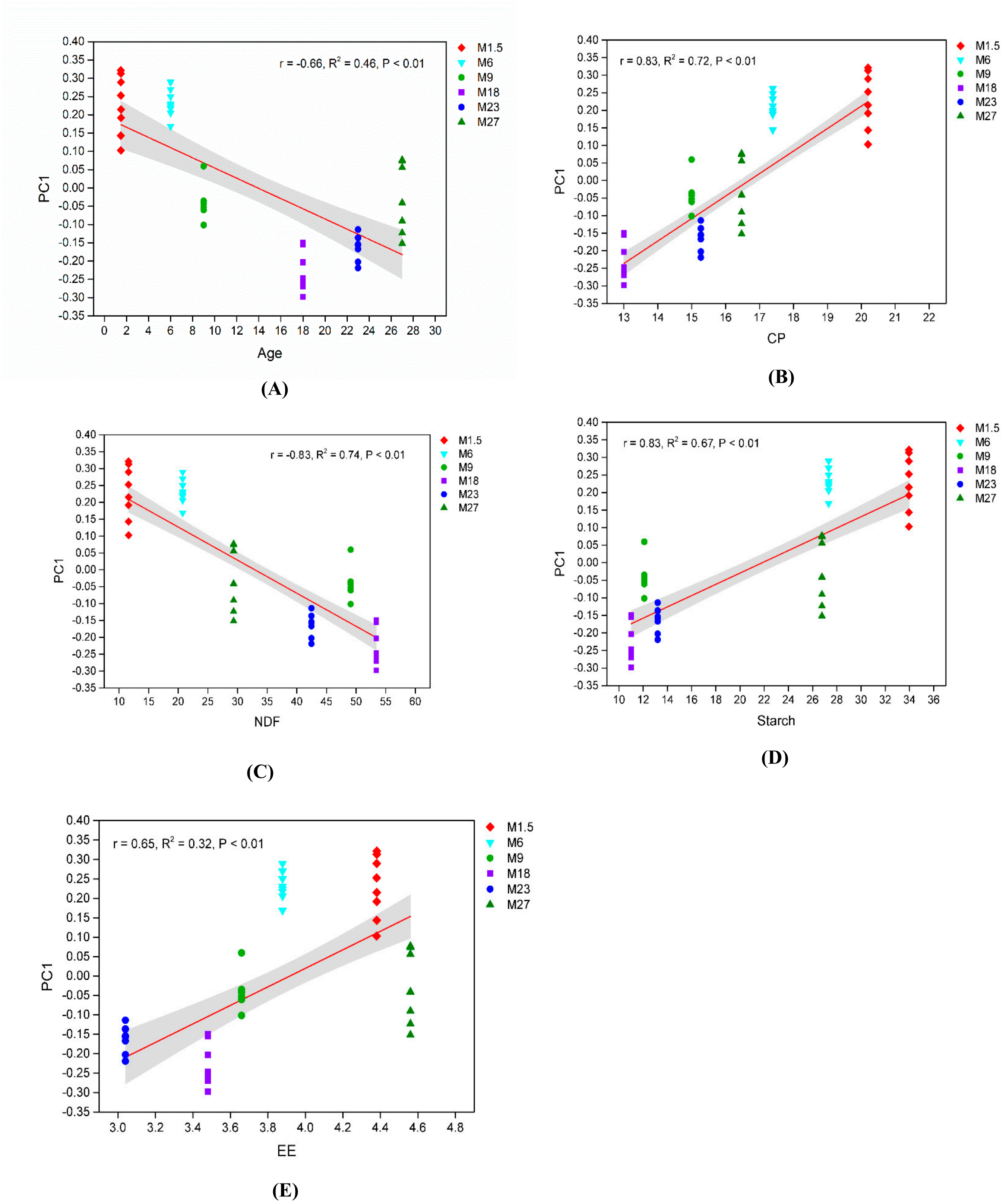

Figure S4. Scatterplot depicting the correlation of (**A**) age, (**B**) CP, (**C**) NDF, (**D**) Starch, and (**E**) EE with the number of PC1. For each sample source, the PC1 values are shown on the y-axis, and the age, CP, NDF, ADF, starch, and EE are on the x-axis. The red line denotes the linear regression line with the gray shading indicating 95% confidence intervals. The Spearman rank correlation (r), Coefficient of determination (R2), and *P*-values are shown at the top of each panel. PC1: Principal component 1 of the principal coordinate analysis' axis of rumen bacteria. CP: crude protein; NDF: neutral detergent fiber; EE: ether extract. M1.5: age of 1.5 months dairy cattle; M6: age of 6 months dairy cattle; M9: age of 9 months dairy cattle; M18: age of 18 months dairy cattle; M23: age of 23 months dairy cattle; M27: age of 27 months dairy cattle.

Table S1. Ingredients and chemical composition of dairy cattle diets (DM basis).

Ingredients	[4]Groups					
	1.5M	6M	9M	18M	23M	27M
[1]Starter	100.0	80				
Whole corn silage			39.4	55.8	59.2	47.7
Oat grass		20	0	0	22.2	2.0
Alfalfa hay			8.3	0	0	7.6
Rice straw			14.5	15.2	0	0
Wheat straw			12.2	10.3	0	0
Extruded soybean meal			0	0	0	1.0
Soybean hulls			0.1	0	0	0.8
Steam flaked corn			1.0	0	1.5	18.8
Spraying corn bran			5.3	7.2	1.7	0
Bran			8.5	6.0	5.0	3.0
DDGS			0	0	4.3	0
Whole cottonseed			0	0	0	3.8
Fat powder			0.4	0	0	0.9
Beet pulp			0.1	0	0	1.3
Sugarcane molasses			0.4	0	0	1.3
Bean pulp			2.0	0	1.5	5.2
Rapeseed meal			3.3	2.0	1.0	3.1
Cottonseed meal			3.5	2.8	2.8	0
[2]Mineral-vitamin premix			1.0	0.7	0.8	3.5
[3]Chemical levels						
CP	20.20	17.42	15.00	13.00	15.27	16.47
EE	4.38	3.85	3.66	3.48	3.04	4.56
Ash	5.43	7.63	10.53	9.92	7.93	9.14
NDF	11.62	20.16	49.11	53.42	42.48	29.34
ADF	4.88	9.98	32.74	34.90	24.51	16.85
Starch	33.95	27.41	12.10	11.02	13.21	26.77
Calcium	0.89	0.76	0.70	0.47	0.32	1.03
Phosphorus	0.50	0.45	0.34	0.30	0.48	0.43

[1] Starter composition: 47.65% Cornmeal, 18.47% Soybean meal, 20.33% Extruded soybean, 5.26% Corn gluten meal, 2.74% Sugarcane molasses, 5.55% Mineral-vitamin premix. [2]1.5, 6, 9, 13, and 18-month-old heifers are fed heifer premix; each Kg of premix (DM basis) contains: vitamin A 279840 IU, vitamin D 27999 IU, vitamin E 1920 IU, iron 144 ppm, manganese 1945 ppm, zinc 2677 ppm, copper 1117 ppm; the 23-month-old heifers are fed prepartum premix, Each Kg premix (DM basis) contains: vitamin A 1249000IU, vitamin D 238261IU, vitamin E 13950IU, iron 15 ppm, manganese 2023 ppm, zinc 2845 ppm, copper 1210 ppm; the 27-month-old heifers are fed lactation premix, per Kg premix (DM basis) contains: vitamin A 176000 IU, vitamin D 4400 IU, Vitamin E 929 IU, manganese 567 ppm, zinc 978 ppm, copper 352 ppm. [3] DDGS: distillers dried grains with soluble; CP: crude protein; EE: ether extract; NDF: neutral detergent fiber; ADF: acid detergent fiber. [4] 1.5M: 1.5-month-old dairy cattle; 6M: 6-month-old dairy cattle; 9M: 9-month-old dairy cattle; 18M: 18-month-old dairy cattle; 18M: 18-month-old dairy cattle; 23M: 23-month-old dairy cattle; 27M: 27-month-old dairy cattle.

Table S2. The number of sequencing reads.

Items	Row Data		Clean Data	
	Seq Number	Base Number	ASVs_Number	Seq_Number
1.5 M	56704	23792867	556d	47271a
6 M	56072	23650864	685c	44650ab
9 M	51122	21471802	1275b	40763b
18 M	52282	21862449	1548a	40700b
23 M	51485	21552178	1504a	40132b

27 M	54283	22817138	1325b	42984ab
Average	53658	22524550	1149	42750
SEM	911	385733	58	773
P values	0.13	0.13	<0.01	0.04

1.5M: 1.5-month-old dairy cattle; 6M: 6-month-old dairy cattle; 9M: 9-month-old dairy cattle; 18M: 18-month-old dairy cattle; 18M: 18-month-old dairy cattle; 23M: 23-month-old dairy cattle; 27M: 27-month-old dairy cattle. Seq: sequence; ASV: amplicon sequence variant; SEM: standard error of mean.

Table S3. The Spearman's correlation coefficient of the top 50 genus bacteria with age or diet (only list the *P* < 0.05 genus).

Genera	Age	CP	NDF	Starch	EE
Succinivibrionaceae_UCG-001	-0.27	0.70**	-.70**	0.70**	0.71**
Succinivibrionaceae_UCG-002	0.61**	-0.67**	.67**	-0.67**	-0.33*
Rikenellaceae_RC9_gut_group	0.13	-0.58**	.58**	-0.58**	-0.48**
unclassified_f_Prevotellaceae	-0.70	0.74**	-.74**	0.74**	0.48**
norank_f_F082	0.69**	-0.76**	0.76**	-0.76**	-0.56**
Succinivibrio	-0.74**	0.62**	-0.62**	0.62**	0.49**
Ruminococcus	0.08	-0.32*	0.32*	-0.32*	-0.43**
norank_f_Muribaculaceae	0.42**	-0.27	0.27	-0.27	-0.14
Prevotellaceae_UCG-001	0.66**	-0.43**	0.43**	-0.43**	-0.33*
Lachnospiraceae_NK3A20_group	-0.78**	0.68**	-0.68**	0.68**	0.38**
Fibrobacter	0.74**	-0.73**	0.73**	-0.73**	-0.51**
norank_f_p-251-o5	0.83**	-0.66**	0.66**	-0.66**	-0.49**
NK4A214_group	0.65**	-0.81**	0.81**	-0.81**	-0.54**
Prevotellaceae_UCG-003	0.72**	-0.76**	0.76**	-0.76**	-0.56**
Christensenellaceae_R-7_group	0.48**	-0.78**	0.78**	-0.78**	-0.57**
unclassified_c_Clostridia	0.65**	-0.82**	0.82**	-0.82**	-0.65**
norank_f_Bacteroidales_RF16_group	0.77**	-0.73**	0.73**	-0.73**	-0.55**
Dialister	-0.59**	0.57**	-0.57**	0.57**	0.43**
Treponema	0.72**	-0.54**	0.54**	-0.54**	-0.07
Saccharofermentans	0.64**	-0.81**	0.81**	-0.81**	-0.66**
Butyrivibrio	-0.17	-0.31*	0.31*	-0.31*	-0.28
Selenomonas	0.33*	0.11	-0.11	0.11	0.33*
norank_f_norank_o_Gastranaerophilales	0.59**	-0.38**	0.38**	-0.38**	-0.39**
norank_f_Bacteroidales_UCG-001	0.74**	-0.80**	0.80**	-0.80**	-0.60**
norank_f_Selenomonadaceae	-0.54**	0.71**	-0.71**	0.71**	0.56**
norank_f_norank_o_Bacteroidales	0.08	-0.17	0.17	-0.17	-0.36*
norank_f_norank_o_Absconditabacteriales_SR1	0.69**	-0.79**	0.79**	-0.79**	-0.52**
Pseudobutyrivibrio	0.65**	-0.84**	0.84**	-0.84**	-0.56**
norank_f_Eubacterium_coprostanoligenes_group	0.51**	-0.65**	0.65**	-0.65**	-0.58**
Ruminobacter	0.32*	-0.28	0.28	-0.28	-0.17
Eubacterium_ruminantium_group	0.78**	-0.60**	0.60**	-0.60**	-0.37**
UCG-004	0.66**	-0.79**	0.79**	-0.79**	-0.60**
norank_f_Lachnospiraceae	0.79**	-0.71**	0.71**	-0.71**	-0.47**
norank_f_UCG-010	0.70**	-0.75**	0.75**	-0.75**	-0.53**
Eubacterium_nodatum_group	-0.84**	0.43**	-0.43**	0.43**	0.15
Roseburia	-0.74**	0.32*	-0.32*	0.32*	0.18
Succinimonas	0.76**	-0.73**	0.73**	-0.73**	-0.57**
Lachnospiraceae_AC2044_group	0.66**	-0.84**	0.84**	-0.84**	-0.58**
Prevotellaceae_UCG-004	0.67**	-0.34*	0.34*	-0.34*	-0.18

"*," and "**," indicate FDR (false discovery rate) adjusted *P*-values <0.05, and <0.01, respectively.
